# Effects of oral anticoagulant therapy in patients with pulmonary diseases

**DOI:** 10.3389/fcvm.2022.987652

**Published:** 2022-08-10

**Authors:** Jiying Lai, Shenghui Feng, Shuo Xu, Xin Liu

**Affiliations:** ^1^Department of Critical Care Medicine, The First Affiliated Hospital of Gannan Medical University, Ganzhou, China; ^2^Queen Mary School, Medical Department, Nanchang University, Nanchang, China; ^3^Department of Respiratory and Critical Care Medicine, The Ganzhou People's Hospital, The Affiliated Ganzhou Hospital of Nanchang University, Ganzhou, China

**Keywords:** direct oral anticoagulants, pulmonary hypertension, pulmonary embolism, pulmonary fibrosis, chronic obstructive pulmonary disease

## Abstract

**Background:**

To evaluate the effect of oral anticoagulants (OACs) therapy, including vitamin K antagonist (VKA) and direct oral anticoagulants (DOAC) in patients with pulmonary diseases.

**Methods:**

Literature from PubMed, MEDLINE, and Cochrane Library were screened until June 2022. Studies assessing OACs for pulmonary hypertension (PH), pulmonary embolism (PE), pulmonary fibrosis (PF), or chronic obstructive pulmonary disease (COPD) were evaluated for inclusion.

**Results:**

Our study indicated that in patients with PH, PE, and COPD, OACs could significantly reduce the mortality risk, and the effects of VKA and DOACs without statistical difference in reducing the risk of recurrent embolism events. In patients with sclerosis-associated pulmonary arterial hypertension (SSc-PAH) or idiopathic pulmonary fibrosis (IPF), vitamin K antagonist (warfarin) significantly increased the mortality risk, while DOACs were not. As for the safety outcome of OACs, existing studies indicate that compared with patients treated with warfarin, the users of DOAC have a lower risk of major bleeding, while there is no statistical significance between them in non-major bleeding events. In current guidelines, the anticoagulation regimen for patients with pulmonary disease has not been defined. The results of our study confirm that DOACs (apixaban, rivaroxaban, dabigatran, and edoxaban) are superior to VKAs in the efficacy and safety outcomes of patients with pulmonary disease.

**Conclusions:**

Oral anticoagulant therapy brings benefits to patients with PH, PE, or COPD, while the anticoagulation regimen for patients with SSc-PAH or IPF requires serious consideration. Compared with VKA, DOAC is a non-inferior option for anticoagulation in pulmonary disease treatment. Further studies are still needed to provide more reliable evidence about the safety outcome of pulmonary disease anticoagulation.

## Introduction

Oral anticoagulants (OACs) bring benefits to patients with a history of atrial fibrillation or flutter, recent major surgery, heart valve replacement, ischemic stroke, and other thrombotic event (1, 2). Common OACs include vitamin K antagonists (VKA, i.e., warfarin) and direct oral anticoagulants (DOACs). The major mechanism of VKA is antagonizing vitamin K, which can inhibit the production of vitamin K involved coagulation factors II, VII, IX and X in the liver. Warfarin has been applied in clinical use for several decades, its indications include the prevention of cardioembolic ischemic stroke, deep venous thrombosis, and pulmonary embolism. However, with a slow onset of action and a narrow therapeutic window, warfarin is closely associated with multiple drug-related life-threatening events. Its anticoagulation effect could be influenced by multiple food and drug interactions: the simultaneous use of warfarin and aspirin, non-steroidal anti-inflammatory drugs (NSAIDs), or clopidogrel will significantly increase the risk of bleeding (3, 4). DOACs directly inhibit coagulation factors Xa and IIa, which do not influence the function of vitamin K. In recent years, DOACs have been confirmed with the function of reducing the risk of stroke and systemic embolism in patients with atrial fibrillation and other artery diseases (5, 6), and they have been approved for the prevention and treatment of venous thromboembolism and systemic and cerebral embolism in patients with atrial fibrillation. Since their anticoagulant effects are more predictable and stable (i.e., less affected by food and drug interactions), the clinical application of DOAC was considered safer than VKA (1).

Pulmonary disease is one of the major threats to human health, which includes pulmonary hypertension (PH), pulmonary embolism (PE), pulmonary fibrosis (PF), and chronic obstructive pulmonary disease (COPD). Pulmonary hypertension is a chronic and progressive disease associated with several cardiovascular conditions, including atrial fibrillation and heart failure. According to distinct mechanisms, PH was divided into five subgroups with similar pathological manifestations and clinical features (7, 8). Lifelong anticoagulation therapy is recommended for patients with pulmonary atrial hypertension (PAH) and chronic thromboembolic pulmonary hypertension (CTEPH), since abnormally high shear stress was identified secondary to excessive vasoconstriction in patients with PAH (9), which could induce vascular remodeling, coagulation cascade derangement, and aberrant platelet function (9). In addition, the decreased fibrinolysis, increased clot formation, endothelium dysfunction, and procoagulant mediators released by platelets could also be detected (9), which significantly increase the risk of thrombosis. CTEPH is characterized by incomplete or abnormal resolution of acute pulmonary embolism, which induces residual emboli to become organized and fibrotic (10). Therefore, it is necessary to use OAC to inhibit thrombosis in patients with PH. As for pulmonary embolism, anticoagulation could reduce mortality by preventing the extension of thrombosis, embolization, and/or formation of new thrombi (11). Associations between idiopathic pulmonary fibrosis (IPF) and many thrombotic vascular diseases, including deep vein thrombosis (DVT), PE, and acute coronary syndromes (ACS) have also been reported by previous studies (12–14). The understanding of IPF etiology remains incomplete, the imbalance between thrombosis and fibrinolysis has been detected in the alveolar compartment in IPF patients, and the systemic pro-thrombotic state might also appear (15). In addition, COPD as one of the most challenging chronic diseases, is closely related to inflammation. Venous thromboembolism (VTE) is a common and potentially fatal complication of COPD, whose morbidity could be significantly increased by COPD (odds ratio between 2 and 9) (16, 17). Moreover, PE is also common comorbidity of COPD (18–20), which might induce disease deterioration. Since all of those pulmonary diseases are associated with vasoconstriction, thrombosis, embolism, or the dysregulation of coagulation (21, 22), OACs have been used for pulmonary disease treatment.

The purpose of this systemic review is to evaluate the existing literature and help clinicians select the appropriate oral anticoagulant regimen for patients with pulmonary diseases.

## Methods

**Figure 1:**
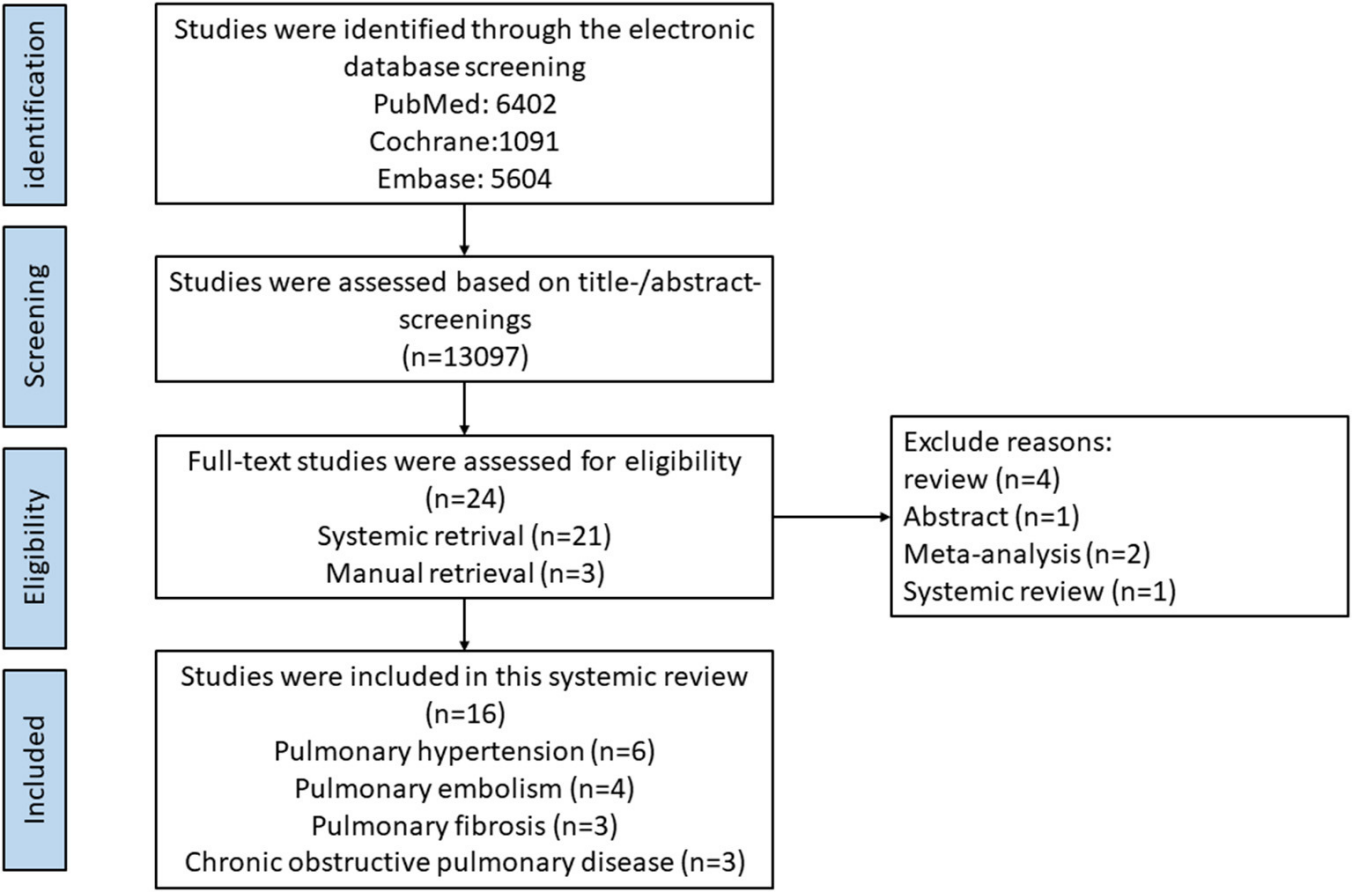
Flow diagram of literature retrieval process of our systemic review.

Two investigators searched electronic databases independently. Relevant articles were screened from PubMed, Embase, and Cochrane Library by using the following keywords: (pulmonary disease OR pulmonary hypertension OR pulmonary fibrosis OR Chronic obstructive pulmonary disease OR pulmonary embolism) AND (oral anticoagulant OR OAC OR vitamin-K antagonist OR VKA OR warfarin OR non-vitamin K oral antagonist OR NOAC OR direct oral antagonist OR DOAC OR apixaban OR rivaroxaban OR edoxaban OR dabigatran OR betrixaban OR novel oral anticoagulant). The last retrieval date was 16th June 2022, and the retrieval process is exhibited in Figure 1.

### Eligibility criteria

Studies were included if they met the following criteria: (1) patients in the study were receiving oral anticoagulants, including vitamin K antagonist warfarin, and non-vitamin K oral anticoagulants such as dabigatran, edoxaban, rivaroxaban, apixaban, or betrixaban; (2) patients with pulmonary disease, including pulmonary hypertension, pulmonary embolism, pulmonary fibrosis, and COPD; (3) case series, case-control studies, cohort studies, or randomized clinical trials were considered to be included in this study; (4) only studies written by English were included in this study; (5) the results were reported as OR, RR or HR value with 95%CI. Specific literature forms including letters, meta-analyses, systemic reviews, cross-sectional studies, reviews, case reports, case series, editorials, and meeting abstracts were excluded in this study. In the pulmonary embolism part, studies investigating venous thromboembolism rather than specific for pulmonary embolism were also considered for exclusion.

### Study selection and data extraction

After screening the titles and abstracts of publications, two authors extracted data independently. Then the full-text screening was conducted to determine whether the literature met the inclusion criteria. Disagreements were resolved by discussing with the third researcher. The baseline information of each study was recorded, including the name of the first author, publication year, the types of anticoagulants, study design, baseline characteristics of the investigated population, and the study outcome.

### Study quality assessment

The quality of eligible cohort studies was assessed by the Newcastle-Ottawa Scale tool, which assesses three aspects of the included studies. The results were marked from 0 to 9 stars, including the cohort selection (0–4 stars), cohort comparability (0–2 stars), and the study outcomes (0–3 stars). Studies with assessment results of <6 stars were considered as low quality. The quality of included randomized controlled trials was evaluated by the Cochrane risk-of-bias tool version 2 for randomized controlled trials. The corresponding results were recorded in Supplementary Tables 1, 2.

## Results

### Pulmonary hypertension

According to ACC/AHA/American College of Chest Physicians guidelines, VKA with the therapeutic INR 1.5–2.5 is suitable for patients with idiopathic PAH (IPAH), and the European Society of Cardiology PH guideline suggests that either VKAs or DOACs could be used as the anticoagulation regimen for CTEPH treatment (23, 24). Totally six investigations evaluated the therapeutic effect of oral anticoagulants in patients with PH were included in our study (Table 1). Only one retrospective cohort study from Sena et al. compared the effect and safety outcomes of warfarin with three different DOACs in patients with CTEPH (25). The rest included studies assessed the therapeutic effect between anticoagulation and non-anticoagulation treatment (26–30), however, whether OAC treatment could increase the risk of bleeding events was not reported in these studies. In most eligible studies comparing warfarin with non-anticoagulation therapy, the administration of warfarin shows non-inferiority. This is consistent with the results of a previous meta-analysis (31). As for the subgroup analysis, four studies reported the efficacy outcome of warfarin in patients with IPAH: two of them indicated that warfarin is not associated with the increased survival (28, 29), while the results of another two studies confirmed the benefits of warfarin treatment (27, 30). Four studies specific for patients with connective tissue disease-associated PAH (CTD-PAH) showed inconsistent results: Ngian et al. (26) reports the benefits of warfarin use in reducing the risk of death, while Olsson et al. and Johnson et al. (29) indicated that there was no statistical difference in terms of survival between warfarin and non-warfarin group in patients with sclerosis-associated pulmonary arterial hypertension (SSc-PAH). In contrast with previous studies, Preston et al. (28) revealed that compared with the non-anticoagulation group, warfarin is associated with poorer survival. A corresponding meta-analysis reported that anticoagulation could significantly reduce the risk of mortality in the overall PAH cohort, the administration of OAC will not increase the mortality risk of CTD-PAH, while it could increase the risk of death in SSc-PAH patients (31). As for the options of OAC, Sena et al. indicated that DOAC shows no inferiority in terms of venous thromboembolism recurrence and mortality. In addition, DOAC and VKA did not exhibit a statistical difference in bleeding events, while DOAC could significantly reduce the risk of major bleeding and the mortality risk according to bleeding events. Collectively, except for SSc-PAH treatment, existing studies tend to indicate that OACs show non-inferiority in reducing mortality risk in patients with PH. Compared with VKA, DOAC is associated with decreased risk of major bleeding, which could be considered as the preference for OAC treatment.

**Table 1:** Summary of included studies of OACs in patients with pulmonary hypertension.

**Author (year)**	**Pulmonary disease**	**Oral anticoagulants**	**Study design**	**Baseline characteristics of investigated population**	**Efficacy outcome**	**Safety outcome**
Sena et al. (2020) (25)	Pulmonary hypertension (CTEPH)	Warfarin, rivaroxaban, dabigatran, apixaba	An observational retrospective study	Chronic thromboembolic pulmonary hypertension Age (mean): 53.54 Female: 50.7% BMI (mean): 28.15	Venous thromboembolism recurrence: warfarin vs. rivaroxaban: 10.1% vs. 8.9% (HR: 1.21, 95% CI, 0.64–2.23; *P* = 0.55). Mortality rates: warfarin vs. rivaroxaban 13.8% vs. 9.7% (HR: 1.61, 95% CI, 0.89–2.99; *P* = 0.11)	Bleeding: warfarin vs. rivaroxaban 27.1% vs. 24.6% (HR: 1.28, 95% CI, 0.86–1.88; *P* = 0.22) Major bleeding: warfarin vs. DOAC (8.9% vs. 14.8%; HR = 1.94, 95% CI = 1.05–3.62, *P* = 0.03) Death according to bleeding events: warfarin vs. rivaroxaban 4.85% vs. 2.2% (HR: 4.75, 95% CI: 1.12–20.16; *P* = 0.03)
Ngian et al. (2012) (26)	Pulmonary hypertension (CTD-PAH)	Warfarin	A cohort study	Patients with right heart catheter proven CTD-PAH	Warfarin therapy: mortality HR = 0.20 (0.05–0.78) *P* = 0.02, 95% CI	NR
Olsson et al. (2014) (27)	Pulmonary hypertension (IPAH, SSc-PAH)	(93%) vitamin K antagonists, heparins (6%) and novel oral anticoagulants (1%).	An observational study	Anticoagulants vs. non-anticoagulants: age: 70 (58–76) vs. 66 (52–75); *P* = 0.001 Female: 64% vs. 63%, *P* = 0.77	Death: IPAH: anticoagulation treatment: HR = 0.79 (0.66–0.94) *P* = 0.007 SSc-PAH: HR = 1.82; 95% CI, 0.94–3.54; *P* = 0.08)	NR
Jonson et al. (2012) (29)	Pulmonary hypertension (SSc-PAH and IPAH)	Warfarin	A cohort study	No warfarin vs. warfarin: SSc-PAH: female: 45 vs. 44%; mean pulmonary artery pressure (mmHg): 38.8 vs. 42.5 IPAH: female: 22% vs. 21%; mean pulmonary artery pressure (mmHg): 51.6 vs. 47.5	Mortality: warfarin vs. no warfarin: SSc-PAH: HR = 1.06 (0.70, 1.63); IPAH: HR = 1.07 (0.57, 1.98)	NR
Preston et al. (2015) (28)	Pulmonary hypertension (SSc-PAH and IPAH)	Warfarin	A cohort study	Warfarin vs. no warfarin: IPAH: age: 50.7 vs. 52.1; female: 80.6% vs. 79.2%; 6MWD: 345.9 vs. 375.1 SSc-PAH: age: 62.8 vs. 64.1; female: 79.1% vs. 90.7%; 6MWD: 290.9 vs. 338.4	Survival: warfarin vs. no warfarin: (adjusted HR) SSc-PAH: HR = 1.60 (0.84–3.06, 95%CI, *P* = 0.15); IPAH: HR = 1.37 (0.84–2.25, 95%CI, *P* = 0.21) SSc-PAH patients receiving warfarin vs. no warfarin in previous 1 year: HR = 1.57; 95% CI, 1.04–2.36; *P* = 0.031) or any time post-baseline HR = 1.49; 95% CI, 1.01–2.20, *P* = 0.046	NR
Kang et al. (2015) (30)	Pulmonary hypertension (IPAH)	Warfarin	A cohort study	Warfarin vs. no warfarin: female: 80% vs. 71.4%; age 32.5 vs. 34.0; 6-MWD (m): 409.0 vs. 451.5	Survival: no warfarin vs. warfarin: OR = 0.210 (0.045–0.976, 95% CI, *P* = 0.047)	NR

*CTD-PAH, connective tissue disease associated pulmonary arterial hypertension; SSc-PAH, sclerosis-associated pulmonary arterial hypertension; IPAH, idiopathic pulmonary arterial hypertension; 6-MWD, 6-min walk distance*.

Sclerosis-associated pulmonary arterial hypertension is the consequence of progressive remodeling of pulmonary vasculature, which is a type of CTD-PAH. It is believed that inflammation and endothelial injury are closely related to SSc-PAH (32). Inflammation is capable of inducing the disequilibrium between vasoactive, proliferative mediators and antiproliferative vasodilators within the endothelium. Under these conditions, pulmonary artery vasoconstriction and cellular proliferation might occur and be exacerbated by platelets releasing serotonin (33). Simultaneously, increased sympathetic excitability, hypoxemia, and ischemia-reperfusion injury of pulmonary vessels promote more cytokine release, which further promote vascular remodeling, fibrosis, and intraluminal microthrombosis (34). Therefore, theoretically, anticoagulation could bring benefits to SSc-PAH patients. However, the outcomes of existing studies are contrary to the theoretical expectations, and the specific reasons are still unclear. Although previous investigations indicated that gastrointestinal vascular lesions seem more commonly in patients with SSc-PAH than IPAH, the increased occurrence of major gastrointestinal bleeding events was not reported in SSc-PAH patients receiving anticoagulants (31). Therefore, the specific reasons for OAC increase the risk of death in patients with SSc-PAH deserves further study, and it is necessary to include more clinical data to further verify the existing research results. In addition, the number of investigations about the safety effects of OAC in PH treatment is still deficient, it is necessary to conduct more relevant experiments to provide reasonable evidence for the choice of anticoagulation regimen in patients with PH.

### Pulmonary embolism

Anticoagulation is also a crucial step for PE treatment. Previous research has indicated that the mortality rate of untreated acute PE has reached 25% (35). The use of anticoagulants is capable of reducing pulmonary embolism-induced mortality rate by preventing the extension of thrombosis, embolization, and/or formation of new thrombi (11). In the past few decades, unfractionated heparin (UFH) and VKAs have been applied to clinics as anticoagulants for PE treatment. After that, since the pharmacodynamic and biological limitations of UFH remain to exist, low-molecular-weight heparins (LMWHs) and the indirect factor Xa (FXa) inhibitor fondaparinux were developed to simplify the management of PE (36). However, the limitations of fondaparinux and VKAs still cannot be eradicated. At present, DOAC is being used in clinics to improve the anticoagulation effect of PE. There are four existing studies reporting the corresponding results, including three randomized trials and one pooled-analysis which pooled the pulmonary embolism-related data from two randomized trials (37–40) (Table 2). Among them, most DOACs have been investigated, including apixaban, edoxaban, rivaroxaban, and dabigatran. The results of existing studies indicate that with the respect to preventing recurrent VTE or VTE-related death, there is no statistical difference between DOACs (including dabigatran, edoxaban, apixaban) and warfarin. As for the safety outcome, two of the included studies reported bleeding events in patients with PE (37, 39). Rivaroxaban and dabigatran could significantly reduce the risk of major bleeding. The risk of first major bleeding or clinically relevant non-major bleeding was only reported in a study focusing on rivaroxaban and warfarin, and the results did not show a statistical difference between them (37). The safety outcome of other DOAC in the PE cohort was not specifically investigated, which deserves further study in the future. In general, current studies support that compared with warfarin, the efficacy of most DOACs shows non-inferiority. In terms of safety outcomes, DOACs significantly reduce the risk of major bleeding. Therefore, they can be used as alternatives to vitamin K antagonists.

**Table 2:** Summary of included studies of OACs in patients with pulmonary embolism.

**Author (year)**	**Pulmonary disease**	**Oral anticoagulants**	**Study design**	**Baseline characteristics of investigated population**	**Efficacy outcome**	**Safety outcome**
The EINSTEIN–PE Investigators	Pulmonary embolism	Rivaroxaban: 15 mg twice daily for the first 3 weeks, followed by 20 mg once daily Edoxaban, warfarin, acenocoumarol: 1.0 mg/kg of body weight twice daily	A randomized, open-label, event-driven, non-inferiority trial	Patients with acute, symptomatic pulmonary embolism with objective confirmation, with or without symptomatic deep-vein thrombosis	Symptomatic recurrent venous thromboembolism: Rivaroxaban vs. standard therapy*: 2.1% vs. 1.8% (HR = 1.12, 95% CI, 0.75–1.68, *P* = 0.003)	First major or clinically relevant non-major bleeding: Rivaroxaban vs. standard therapy: 10.3% vs. 11.4% (HR = 0.90; 95% CI, 0.76–1.07; *P* = 0.23) Major bleeding: Rivaroxaban vs. standard therapy: 1.1% vs. 2.2%, (HR = 0.49; 95% CI, 0.31–0.79; *P* = 0.003)
Goldhaber et al. (2016) (39)	Pulmonary embolism	Warfarin (therapeutic INR range, 2.0–3.0), dabigatran 150 mg twice daily for 6 months (double-dummy, ‘oral-only' treatment period).	A pooled analysis	Dabigatran vs. warfarin: age: 55.6 vs. 55.6; male: 52.8% vs. 53.4%	In patients with PE, recurrent VTE/VTE-related death: dabigatran vs. warfarin: 2.9 % vs. 3.1 % (HR = 0.93, 0.53–1.64, *P* = 0.4848)	Major bleeding: dabigatran vs. warfarin HR= 0.60, 0.36–0.99
The Hokusai-VTE Investigators	Pulmonary embolism	Edoxaban: 60 mg once daily, or 30 mg once daily; warfarin	A randomized, double-blind, non-inferiority study	Edoxaban vs. warfarin: age: 57.1 vs. 57.4; male: 52.3% vs. 52.4%	First recurrent VTE or VTE-related death: Edoxaban vs. warfarin: 2.8% vs. 3.9%, HR = 0.73 (0.50–1.06), 95%CI	NR
Agnelli et al. (2013) (40)	Pulmonary embolism	Apixaban group: 10 mg of apixaban twice daily for the first 7 days, followed by 5 mg twice daily for 6 months Conventional therapy: enoxaparin 1 mg/kg of body weight every 12 h for at least 5 days. Warfarin: INR between 2.0 and 3.0	A randomized, double-blind study	Apixaban vs. conventional therapy: age: 57.2 vs. 56.7; male: 58.3% vs. 59.1%	Recurrent symptomatic venous thromboembolism or death related to venous thromboembolism: apixaban vs. conventional group: 2.3% vs. 2.6%, RR = 0.90; 95% CI, 0.50–1.61	NR

### IPF

Idiopathic pulmonary fibrosis is a type of fatal disease, whose 5-year survival is even worse than many cancers. The understanding of IPF etiology remains incomplete, and its diagnosis often requires the cooperation of multidisciplinary teams (46, 47). Associations between IPF and many thrombotic vascular diseases, including deep vein thrombosis (DVT), pulmonary embolism, and acute coronary syndromes (ACS) have been reported in previous studies (12–14). In addition, the imbalance between thrombosis and fibrinolysis has been detected in the alveolar compartment in IPF patients, and the systemic pro-thrombotic state could also appear in IPF patient (15). Therefore, theoretically, the application of oral anticoagulants could improve the therapeutic effect of IPF, and the benefits of warfarin use were reported in previous randomized trials (41, 48). After literature screening, three studies meet the inclusion criteria: the first one is a randomized trial, which compared warfarin and placebo in IPF patients (Table 3). The corresponding results showed that warfarin was associated with increased mortality risk in the IPF population lacking other indications for anticoagulation (41). The second study detected the influence of mortality and transplantation in the non-anticoagulation group and patients using warfarin or DOAC for anticoagulation (22). The adjusted result indicated that warfarin is associated with increased mortality and reduced transplant-free survival, while DOACs were not. Then the result of the third study compared the efficient outcome of DOAC and warfarin, which confirmed the advantages of DOAC in reducing the mortality risk in IPF patients (42). All of these three studies did not evaluate the safety outcome of oral anticoagulation in IPF treatment. Collectively, warfarin is associated with an increased mortality risk in IPF patients and DOACs seem more suitable for their anticoagulation treatment.

**Table 3:** Summary of included studies of OACs in patients with pulmonary fibrosis.

**Author (year)**	**Pulmonary disease**	**Oral anticoagulants**	**Study design**	**Baseline characteristics of investigated population**	**Efficacy outcome**	**Safety outcome**
Noth et al. (2012) (41)	Pulmonary fibrosis	Study subjects were provided two strengths of warfarin tablets (1 and 2.5 mg) or matching placebos	Randomized trial	Patients aged 35–80 years with progressive Idiopathic pulmonary fibrosis Warfarin vs. placebo: age: 67.3 vs. 66.7; Female: 33% vs. 21%; FVC % predicted: 58.9 vs. 58.7	Primary outcome: the composite outcome of time to death, hospitalization (non-bleeding, non-elective), or a 10% or greater absolute decline in FVC: warfarin vs. placebo: HR = 1.32 (0.70, 2.47), *P* = 0.271 All-cause mortality: warfarin vs. placebo: HR = 4.85 (1.38, 16.99), *P* = 0.005 Combined all-cause mortality or non-elective, nonbleeding hospitalizations: warfarin vs. placebo: HR = 2.12 (1.00, 4.52), *P* = 0.02 Combined all-cause mortality or >10% FVC drop: warfarin vs. placebo: HR = 1.44 (0.69, 2.99), *P* = 0.28	NR
Naqvi et al. (2021) (42)	Pulmonary fibrosis	Warfarin or DOACs including apixaban, rivaroxaban, or dabigatran	A retrospective cohort study	Patients with IPF, warfarin vs. DOAC: age 73.29 vs. 74.09; male: 50% vs. 57.8%; atrial fibrillation: 57.1% vs. 64.4%; CHA_2_DS_2_-VASc (median, IQR): 4 (1.5) vs. 4 (1.25); Thromboembolism: 28.6 vs. 35.6%; Inherited coagulopathy with thrombotic event: 10.7% vs. 2.2%	One year follow-up of mortality: DOAC vs. warfarin: OR = 77.4, 95% CI, 5.94–409.3, *P* = 0.007	NR
King et al. (2021) (22)	Pulmonary fibrosis	Warfarin, DOACs (apixaban, rivaroxaban, dabigatran)	Cohort study	Patients with Interstitial Lung Disease in the Pulmonary Fibrosis Foundation Patient Registry DOAC vs. warfarin vs. none: age: 70.45 vs. 70.40 vs. 67.37; Male: 73.1% vs. 71.6% vs. 62.2%; BMI: 29.48 vs. 30.22 vs. 29.35	Reduced transplant-free survival: (adjusted data) warfarin (HR = 2.566; 95% CI, 1.095–6.0165, *P* = 0.014); DOACs (HR = 1.368; 95% CI, 0.500–3.737)	NR

Warfarin interferes with the metabolism of vitamin K, disturbing the production of carboxylated vitamin K-dependent clotting factors. In addition, vitamin K is also essential for the production of the endogenous anticoagulant protein C (46). The administration of warfarin could induce the deficiency of protein C before vitamin K-dependent clotting factors depletion, leading to a transient procoagulant state (46). Protein C can also alter the expression level of inflammatory and apoptotic genes, down-regulate the release of inflammatory mediators, reduce the expression of cell adhesion molecules and maintain the barrier function of endothelial cells (49). Interference with these protective pathways might be the reason for worse outcomes in IPF treatment. However, the major difference between warfarin and DOAC is their influence on vitamin K, there is no existing study reporting whether the function of vitamin K will affect the survival of IPF, which deserves more attention in the future.

### COPD

Chronic obstructive pulmonary disease is associated with neutrophilic inflammation and T-lymphocytes activation (17). In recent years, macrophages have also been confirmed to be involved in COPD: the inhaled particles activate alveolar macrophages, which then release cytokines and chemokines, including interleukins (IL)-1α, IL-1β, IL-33, and IL-18 (50). These cytokines inhibit plasminogen activators. The procoagulant effect is initiated by tissue factor, IL-6, and the tumor necrosis factor (17). All of the above cytokines contribute to the amplification of procoagulant processes, therefore, COPD could induce the occurrence of venous thromboembolism and pulmonary embolism. A previous multicentral cross-sectional study indicated that PE could be detected in 5.9% of patients with COPD (19), and the correlation between COPD and the risk of embolism might be the basis of its poor prognosis (51). Previous studies have confirmed the effect of anticoagulation in COPD treatment: improved lung function has been detected in patients hospitalized for COPD deterioration and treated with low molecular weight heparin (LMWH). After anticoagulation treatment, FEV1, and PaO_2_ were increased and PaCO_2_ was decreased, and therefore the reduction of D-Dimers and blood clotting parameters was observed (52). Anticoagulation is capable of improving the hemorheological indexes, circulatory and pulmonary functions, which can also reduce patients' blood viscosity.

As for OAC treatment in patients with COPD, totally three studies were included in our systemic review (43–45) (Table 4). All of them indicated that the use of oral anticoagulants could reduce the mortality risk of COPD, and the efficacy outcome between apixaban and warfarin did not exhibit statistical difference. The safety outcome of OAC in COPD treatment was only reported by Durheim et al. in 2018 (45), which indicated that compared with no anticoagulation, OAC will not increase the risk of major bleeding. Considering that current reported data about the efficacy and safety of OAC in COPD treatment are relatively limited, in future research, the safety outcome of different OACs deserves special attention, which could provide suggestions for clinical medication.

**Table 4:** Summary of included studies of OACs in Patients with COPD.

**Author (year)**	**Pulmonary disease**	**Oral anticoagulants**	**Study design**	**Baseline characteristics of investigated population**	**Efficacy outcome**	**Safety outcome**
Durheim et al. (2016) (43)	Atrial fibrillation, COPD	Apixaban, warfarin	Insights of the results of prospective, multi-center cohort study	NR between apixaban and warfarin groups	stroke or systemic embolism: Apixaban vs. warfarin: HR = 0.92 [95% CI 0.52–1.63] All-cause mortality: apixaban vs. warfarin: HR = 0.80 [95% CI 0.62–1.04]	NR
Andersson et al. (2019) (44)	COPD, right-sided heart failure	Warfarin (96%), DOAC (4%)	Cohort study	NR between patients treated with or without oral anticoagulants	Death: oral anticoagulants treatment vs. no oral anticoagulants treatment: HR = 0.88 (0.85–0.92, 95% CI)	NR
Durheim et al. (2018) (45)	COPD	OAC (warfarin and dabigatran)	Cohort study	NR between OAC and no OAC groups	All cause death: OAC vs. no OAC: HR = 0.77 (95% CI; 0.59–1.01) Cardiovascular death: OAC vs. no OAC: HR = 0.76 (95% CI; 0.49–1.18) Non-cardiovascular death: OAC vs. no OAC: HR = 0.81 (95% CI; 0.58–1.15) 1st cardiovascular hospitalization: OAC vs. no OAC: HR = 0.97 (95% CI; 0.79–1.21)	1^st^ major bleed: OAC vs. no OAC: HR = 1.22 (95% CI; 0.84–1.75) 1st bleeding hospitalization: HR = 1.12 (95% CI; 0.72–1.66)

## Future directions

Totally 16 studies were included in our systemic review to present the efficacy and safety outcomes of OAC therapy in patients with pulmonary diseases. However, in terms of the selection of anticoagulation regimen, multiple crucial clinical questions remain uncertain. First of all, existing investigations have shown that the use of OAC will increase the risk of death in patients with SSc-PAH. However, the relevant theoretical mechanism is still unclear, and more clinical data need to be included in the analysis to confirm the reliability of this conclusion. Secondly, the results of our study indicated that warfarin is associated with increased mortality risk in patients with IPF, while DOACs were not. Considering that the major difference between VKA and DOAC is the action of vitamin K, it is reasonable to suspect that vitamin K plays a role in reducing the risk of death in IPF patients. However, existing studies can only provide some theoretical evidence about this assumption, there is no study reporting the relationship between Vitamin K and IPF survival, which deserves further investigation. Thirdly, the results about the safety effect of OAC in pulmonary diseases are still very limited. Our conclusion is based on the evaluation of the corresponding results in patients with PH and PE. The risk of OAC treatment-associated bleeding events specific to IPF patients has not been reported in the included studies, and there are only comparison results between anticoagulation and non-anticoagulation groups in COPD population. It is necessary to investigate more about the safety outcome of OAC in patients with different pulmonary diseases in the future. In addition, obtaining more data comparing the safety results of VKA and DOAC in different pulmonary diseases will help to provide more accurate recommendations for the selection of clinical anticoagulation regimen. Finally, included studies in our systemic review only reported the corresponding results of apixaban, dabigatran, edoxaban, and rivaroxaban, none of them assessed the effects of betrixaban in patients with pulmonary diseases. Completely investigating all types of DOAC will be more conducive to accurately evaluate its effect in the treatment of pulmonary diseases.

## Conclusion

Oral anticoagulant (including VKA and DOAC) could significantly reduce the mortality risk in patients with PH, PE, and COPD, and the effects of DOAC in mortality-reducing and VTE recurrence preventing are no less than warfarin. In patients with IPF or SSc-PAH, warfarin could significantly increase the mortality risk and reduce the transplant-free survival, while DOACs are not. Compared with warfarin, DOACs show non-inferiority in the bleeding events, and they can also significantly reduce the risk of major bleeding. DOAC therapy should be regarded as a non-inferior option for stroke and embolism prevention in patients with pulmonary diseases.

## Data availability statement

The original contributions presented in the study are included in the article/Supplementary material, further inquiries can be directed to the corresponding authors.

## Author contributions

All authors listed have made a substantial, direct, and intellectual contribution to the work and approved it for publication.

## Conflict of interest

The authors declare that the research was conducted in the absence of any commercial or financial relationships that could be construed as a potential conflict of interest.

## Publisher's note

All claims expressed in this article are solely those of the authors and do not necessarily represent those of their affiliated organizations, or those of the publisher, the editors and the reviewers. Any product that may be evaluated in this article, or claim that may be made by its manufacturer, is not guaranteed or endorsed by the publisher.
