# Supplementary material for: Effects of oral anticoagulant therapy in patients with pulmonary diseases
Source: Front Cardiovasc Med. 2022 Aug 10;9:987652. doi: 10.3389/fcvm.2022.987652 (PMC9399807; doi:10.3389/fcvm.2022.987652)
Supplement: Supplementary file 1 [file Table_1.DOCX]

| **Supplemental Table 1 Quality of the cohort studies included in the systemic review assessed by Newcastle-Ottawa quality assessment (NOS) scale** | | | | | | | | | |
| --- | --- | --- | --- | --- | --- | --- | --- | --- | --- |
|  | Selection | | | | Comparability | Outcome | | |  |
| Cohort Study | Representativeness of the exposed cohorts | Selection of the non-exposed cohort | Ascertainment of exposure | Demonstration that outcome of interest was not present at start of study | Comparability of cohorts on the basis of the design or analysis | Assessment of outcome | Was follow-up long enough for outcomes to occur | Adequacy of follow up of cohorts | Quality score |
| Sena et al. (2020) | ★ | ★ | ★ |  | ★★ | ★ | ★ | ★ | 8 |
| Ngian et al. (2012) | ★ | ★ | ★ | ★ | ★★ | ★ | ★ | ★ | 9 |
| Olsson et al. (2013) | ★ | ★ | ★ | ★ | ★★ | ★ | ★ |  | 8 |
| Jonson SR et al (2012) | ★ | ★ | ★ | ★ | ★★ | ★ |  |  | 7 |
| Preston et al (2015) | ★ | ★ | ★ | ★ | ★★ | ★ | ★ |  | 8 |
| Kang et al (2015) | ★ | ★ | ★ | ★ | ★★ | ★ | ★ | ★ | 9 |
| King et al. (2021) | ★ | ★ | ★ | ★ | ★★ | ★ |  |  | 7 |
| Naqvi et al. (2021) | ★ | ★ | ★ | ★ | ★★ | ★ | ★ | ★ | 9 |
| Durheim et al. (2016) | ★ | ★ | ★ |  | ★★ | ★ | ★ | ★ | 8 |
| Durheim et al. (2018) | ★ | ★ | ★ |  | ★★ | ★ | ★ |  | 7 |
| Andersson et al. (2019) | ★ | ★ | ★ |  | ★ | ★ | ★ | ★ | 7 |

**Supplemental Table 2 Quality of Randomized clinical trials included in this systemic review assessed by version 2. Cochrane Risk of Bias Tool**

| **Study title** | **Randomization process** | **Deviations from the intended intervention** | **Missing outcome data** | **Measurement of the outcome** | **Selection of the reported result** | **Overall risk of bias** |
| --- | --- | --- | --- | --- | --- | --- |
| **EINSTEIN–PE** | Low risk | Low risk | Low risk | Low risk | Low risk | Low risk |
| **Hokusai** | Low risk | Low risk | Low risk | Low risk | Low risk | Low risk |
| **AMPLIFY** | Low risk | Low risk | Low risk | Low risk | Low risk | Low risk |
| **ACE-IPF** | Low risk | Low risk | Low risk | Low risk | Low risk | Low risk |
